# Abnormal Changes of Monocyte Subsets in Patients With Sjögren’s Syndrome

**DOI:** 10.3389/fimmu.2022.864920

**Published:** 2022-03-04

**Authors:** Yan He, Rongjuan Chen, Mengqin Zhang, Bin Wang, Zhangdi Liao, Guixiu Shi, Yan Li

**Affiliations:** ^1^Department of Rheumatology and Clinical Immunology, The First Affiliated Hospital of Xiamen University, School of Medicine, Xiamen University, Xiamen, China; ^2^Xiamen Key Laboratory of Rheumatology and Clinical Immunology, Xiamen Science and Technology Bureau, Xiamen, China

**Keywords:** Sjögren’s syndrome, monocyte subsets, single cell RNA-sequencing, pathogenesis, transcriptomic analyses

## Abstract

**Background:**

Recent studies have proven the existence of distinct monocyte subsets, which play a significant role in the development of some rheumatic diseases such as systemic lupus erythematosus (SLE). This study was performed to define the changes of monocyte subsets in patients with Sjögren’s Syndrome (SjS).

**Methods:**

Single cell RNA-sequencing (scRNA-seq) data of monocytes from SjS patients and controls were analyzed. The transcriptomic changes in monocyte subsets between SjS and controls were identified and potential key functional pathways involved in SjS development were also explored.

**Results:**

A total of 11 monocyte subsets were identified in the scRNA-seq analyses of monocytes. A new monocyte subset characterized by higher expression of VNN2 (GPI-80) and S100A12 (Monocyte cluster 3) was identified, and it was increased in SjS patients. Compared with controls, almost all monocyte subsets from SjS patients had increased expression of TNFSF10 (TRAIL). Moreover, interferon (IFN)-related and neutrophil activation-associated pathways were main up-regulated pathways in the monocytes of SjS patients.

**Conclusion:**

This study uncovered the abnormal changes in monocyte subsets and their transcriptomic changes in SjS patients, and identified TNFSF10 ^high/+^ monocytes as a potential key player in SjS pathogenesis and a promising target for SjS treatment.

## Introduction

SjS is a complex rheumatic disease characterized by the infiltration of immune cells into exocrine glands such as salivary glands, and effective targeted therapies for SjS are still lacking (1, 2). Current studies suggest that some factors such as disease susceptibility genes, immune abnormalities, and viral infections are synergistically involved in its pathogenesis of SjS (3–5). Among those factors, abnormal immune factors such as B cell hyperactivity have been considered as key players in SjS pathogenesis and potential targets for SjS treatment (6, 7). Nevertheless, the immune mechanisms involved in SjS pathogenesis and progression have not been fully clarified. To reveal potential targets of immunotherapy, it is necessary to further study the immune cell subsets that play a critical pathogenic role in SjS.

Mononuclear phagocytes (MNPs) are the most common innate immune cells with key roles in both immunity and autoimmunity (8–11). MNPs in blood are mainly composed of monocytes and dendritic cells (DCs), both of which have heterogenerous subsets with distinct phenotypes (12, 13). Recent studies using scRNA-seq have demonstrated the existence of distinct monocyte subsets and they have crucial roles in the development of some rheumatic diseases such as SLE (14, 15). For instance, monocytes can participate in the pathogenesis of SLE by immune mechanisms such as secreting pro-inflammatory cytokines and assisting in the activation of B cells and T cells (16–19). In recent years, the roles of monocytes in the pathogenesis of SjS have also gained increased attentions, and some studies have proved possible key roles of monocytes in the development and progression of SjS (20–24). However, the mechanisms of monocytes in SjS have not been fully clarified, and further research is required. At present, there is a lack of relevant research exploring the changes of monocyte subsets in SjS patients *via* scRNA-seq. This study aimed to analyze the abnormal changes of monocyte subsets in peripheral blood of SjS patients by scRNA-seq data, and further explore the key transcriptomic changes in monocytes of SjS patients.

## Materials and Methods

### Transcriptomic Data of Monocytes of SjS Patients

scRNA-seq data of monocytes from SjS patients and controls in GSE157278 from Gene Expression Omnibus (GEO) were used in our study. In GSE157278, peripheral blood mononuclear cells (PBMCs) from 5 SjS patients and 5 controls were analyzed by scRNA-seq, but this study did not analyze the abnormal changes of monocyte subsets in peripheral blood of SjS patients by scRNA-seq analyses. In addition, a sample with low quality of sequencing was further excluded. Therefore, we analyzed scRNA-seq data of monocytes from 5 SjS patients and 4 controls. This study was conducted in accordance with the Declaration of Helsinski and was approved by the Ethics Committee of our hospital.

To further explore the transcriptomic changes in monocytes of SjS patients, we further analyzed bulk RNA-sequencing (RNA-seq) data of monocytes of SjS patients in GSE173670. In GSE173670, RNA-sequencing of CD14^+^ monocytes from SjS patients and controls was carried out. We analyzed the transcriptomic changes in monocytes of 12 SjS patients and 11 healthy controls.

### scRNA-Seq Analyses

scRNA-seq analyses were performing using Seurat (Version 3.0) and SingleR (25, 26). Quality control was performed mainly by the amount of feature genes and the percentage of mitochondrial genes expression. To characterize the subsets of monocytes precisely, scRNA-seq data with high quality were analyzed. Cells with detected genes above 1000 and the percentage of mitochondrial genes less than 10% were regarded as cells with high quality. Cells were omitted if they were more than 10% in the percentage of mitochondrial genes expression. Monocytes in each sample were identified by SingleR and dentritic cells were filtered (25), in which up to 15 principal components (PCs) were used in the clustering of cells. Gene counts were normalized with SCTransform function of Seurat. Intergraded data from 5 SjS patients and 4 controls were clustered with 9 PCs in combination with the dimensional reduction method of uniform manifold approximation and projection (UMAP) or t-Distributed Stochastic Neighbor embedding (t-SNE). Cell type annotation was performed with SingleR and those cells annotated to be monocytes were extracted for subsequent analyses. Feature genes of monocyte subsets were calculated through the differential expression analyses in Seurat.

### Differential Expression Analyses

In the analyses of RNA-seq transcriptome datasets, gene expression analyses with raw count were first used if available, and differential expression analyses were performed with DESeq2 (27). For RNA-seq transcriptome datasets in other data forms such as FPKM (Fragments Per Kilobase Million) or TPM (Transcripts Per Million), differential expression analyses were performed with limma package (28). In the differential gene expression analyses above, outcome lists of differentially expressed genes (DEGs) were obtained for subsequent analyses. Those genes with the log2 value of fold changes (log2FC) no less than 1 and adjusted P values less than 0.05 were deemed to be DEGs. In scRNA-seq analyses, DEGs of monocyte subsets between SjS patients and controls were calculated through the differential expression analyses in Seurat.

### Enrichment Analyses of DEGs

Functional annotation of the DEGs was performed with clusterProfiler (29), and gene sets of gene ontology (GO) terms and hallmark gene sets were mainly analyzed in the functional enrichment analyses. Genes sets with an adjusted P values less than 0.05 were considered as significantly enriched pathways.

### Expression of Key Genes in SjS Patients

The aberrant expression of potential key genes in the monocytes of patients was validated with the transcriptomic data from 12 SjS patients and 11 healthy controls in GSE173670. The expression levels of potential key genes were extracted, and difference between SjS patients and controls was then analyzed.

### Statistical Analyses

Results were shown as mean or median with 95% confidence intervals (95%CI). The difference in the expression levels of potential key genes between SjS cases and controls was analyzed with the unpaired t test. R software (Version 3.6.1) and GraphPad Prism (Version 8) were used in data analyses and P<0.05 suggested statistically significant difference. An online software (http://www.openepi.com/) was used in the power calculation. As the expression values in the transcriptomic data of GSE173670 had been standardized and inter-sample variability was small, the pre-defined standard deviation was 0.85. For a gene with a difference of 1.0 in the expression level, a sufficient power over 80% needed a combined set of 12 cases and 12 controls. In the *post-hoc* power analyses, the power of detecting a statistical significant difference in TNFSF10 (TRAIL) expression level between cases and controls was 95.5%.

## Results

### scRNA-Seq Analyses Revealed Changes in Monocyte Subsets Among SjS Patients

**Figure 1:**
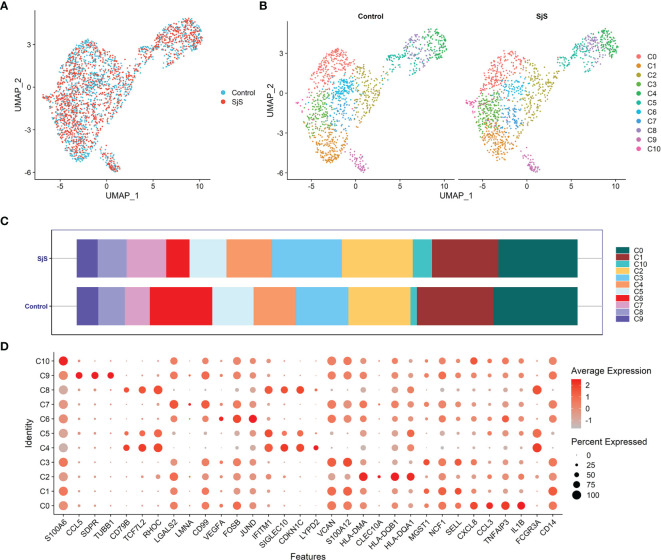
Identification of monocyte subsets among SjS patients *via* scRNA-seq transcriptome analyses. **(A)**, Visualization of the distribution of monocytes between SjS patients and controls *via* the UMAP dimension reduction method. **(B)**, Visualization of clusters of monocytes between SjS patients and controls *via* the UMAP dimension reduction method. **(C)**, Comparison of the percentages of monocyte clusters between SjS patients and controls. **(D)**, Dot plot shows the expression percentages and the expression levels of feature genes of different clusters of monocytes.

In the scRNA-seq analyses of monocytes of SjS patients, a total of 11 monocyte subsets were identified (Cluster 0 to Cluster 10; shown as C0 to C10 in **Figure 1**). A new monocyte subset characterized by higher expression of VNN2 (GPI-80) and S100A12 (Monocyte cluster 3, C3) was identified, and it was increased in SjS patients (**Figures 1B, C**). However, owing to the limited samples in current study, the feature genes of monocyte subsets were not highly specific, and were also expressed in other subsets (**Figure 1D**). The changes in monocyte subsets among SjS patients still need to be explored by scRNA-seq analyses of larger number of samples.

### Transcriptomic Changes in Monocytes of SjS Patients

**Figure 2:**
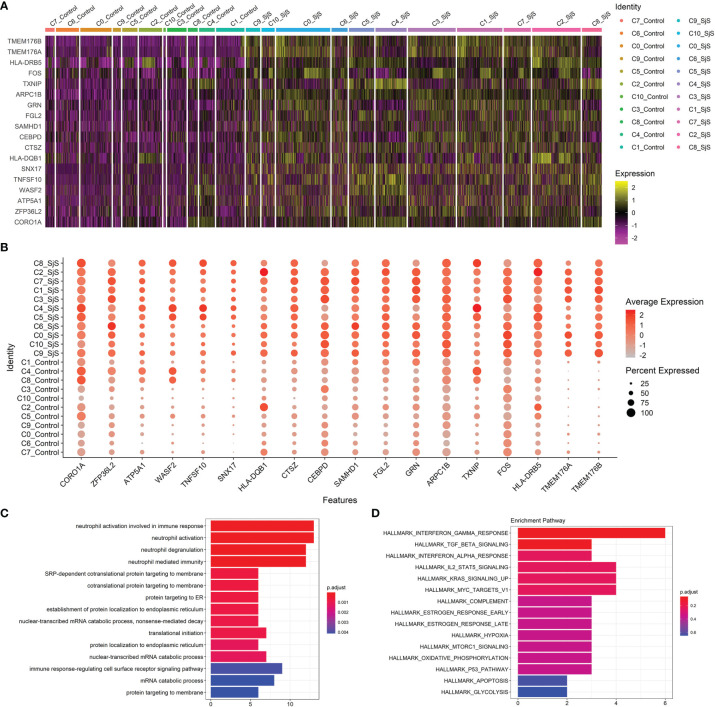
Identification of transcriptomic changes in monocyte subsets of SjS patients *via* scRNA-seq transcriptome analyses. **(A)**, Heatmap shows the expression changes of key genes in those monocyte subsets between SjS patients and controls. **(B)**, Dot plot shows the expression percentages and the expression levels of key DEGs of monocyte subsets between SjS patients and controls. **(C)**, Main enriched GO pathways of those significant DEGs identified in scRNA-seq transcriptome analyses *via* clusterProfiler. **(D)**, Main enriched Hallmark pathways of those significant DEGs identified in scRNA-seq transcriptome analyses *via* clusterProfiler.

Through scRNA-seq transcriptome analyses, a number of significant DEGs in monocyte subsets of SjS patients were identified, such as TMEM176B, TMEM176A, HLA-DRB5, FOS, TXNIP, ARPC1B, GRN, FGL2, SAMHD1, CEBPD, CTSZ, HLA-DQB1, SNX17, TNFSF10, WASF2, ATP5A1, ZFP36L2 and CORO1A (**Figures 2A, B**). Some of those significant DEGs above such as HLA-DRB5 and TNFSF10 have been proved to be key players in the pathogeneses of many autoimmune or rheumatic diseases. Enrichment analyses of those significant DEGs identified neutrophil activation-associated pathways and IFN-related pathways as the main up-regulated pathways in the monocytes of SjS patients, (**Figures 2C, D**) suggesting that those pathways had the vital roles in SjS pathogenesis.

**Figure 3:**
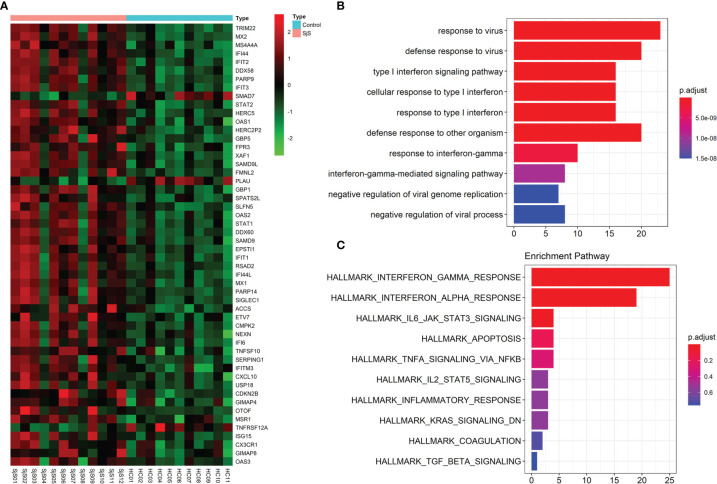
Bulk transcriptome analyses of monocytes identify key genes and functional pathways involved in SjS. **(A)**, Top DEGs in the bulk transcriptome analyses of monocytes from SjS patients. **(B)**, Main enriched GO pathways of those significant DEGs *via* clusterProfiler. **(C)**, Main enriched Hallmark pathways of those significant DEGs *via* clusterProfiler.

Bulk transcriptome analyses of monocytes identified a number of significant genes aberrantly expressed in the monocytes of SjS patients such as TRIM22, MX2, MS4A4A, IFI44, IFIT2, STAT2, SAMD9L, STAT1, EPSTI1, IFI44L, SIGLEC1, TNFSF10, CX3CR1 and ISG15 (**Figure 3A**). Enrichment analyses of those significant DEGs suggested that those genes were enriched in the pathways such as virus infection-associated and IFN-related pathways (**Figures 3B, C**), indicating that those pathways played a key role in the pathogenesis of SjS.

### Increased Expression of TNFSF10 (TRAIL) in the Monocytes of SjS Patients

**Figure 4:**
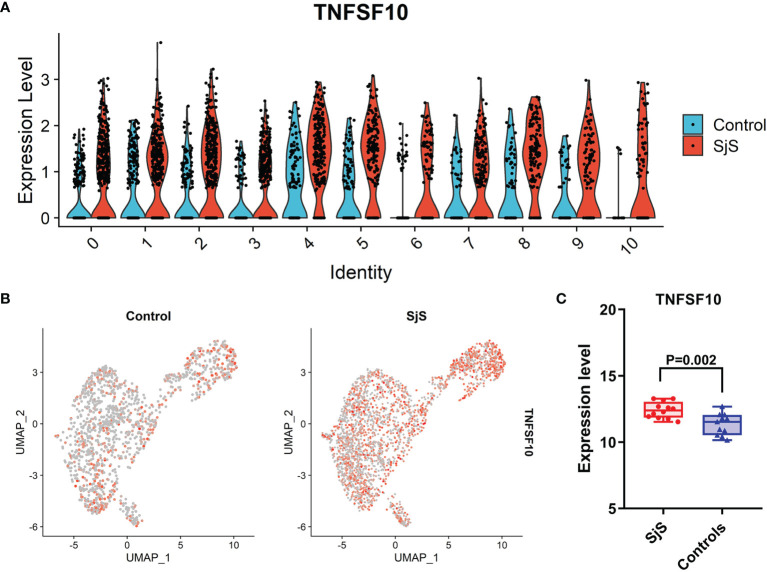
Increased expression of TNFSF10 (TRAIL) in monocytes of SjS patients. **(A)**, Violin plot shows the increased expression of TNFSF10 (TRAIL) in most monocyte sunsets of SjS patients in the scRNA-seq transcriptome analyses. **(B)**, Feature plot shows the increased expression of TNFSF10 (TRAIL) in monocytes of SjS patients in the scRNA-seq transcriptome analyses. **(C)**, Validation study confirms the increased expression of TNFSF10 (TRAIL) in monocytes of SjS patients.).

Among those significant DEGs, the increased expression of TNFSF10 (TRAIL) in the monocytes of SjS patients was identified by both scRNA-seq transcriptome analyses (**Figure 2B**) and bulk transcriptome analyses of monocytes (**Figure 3A**). As shown in **Figures 4A, B**, the increased expression of TNFSF10 (TRAIL) was found in most monocyte subsets of SjS patients. In addition, validation study also confirmed the increased expression of TNFSF10 (TRAIL) in monocytes of SjS patients (**Figure 4C**). The outcomes above suggested TNFSF10 ^high/+^ monocytes as a potential key player in SjS pathogenesis and a promising target for SjS treatment.

## Discussion

The roles and underlying mechanisms of monocytes in SjS development have not been fully clarified. This study analyzed the abnormal changes of monocyte subsets in SjS patients by scRNA-seq data, and further explored the key transcriptomic changes of monocytes in SjS patients. We found a new monocyte subset characterized by higher expressions of VNN2 (GPI-80) and S100A12 (Monocyte cluster 3), which was increased in SjS patients. Moreover, virus infection-associated pathways, IFN-related pathways and neutrophil activation-associated pathways were the major up-regulated pathways in the monocytes of SjS patients. Finally, compared with controls, almost all monocyte subsets from SjS patients had increased expression of TNFSF10 (TRAIL). Therefore, this study uncovered the abnormal changes in monocyte subsets and their transcriptomic changes in SjS patients, and identified TNFSF10 ^high/+^ monocytes as a potential key player in SjS pathogenesis and a promising target for SjS treatment.

Mononuclear phagocytes including monocytes are the main antigen presenting cells (APCs) and can initiate protective immune processes against pathogens, but they can also initiate autoimmune processes in autoimmune diseases (30–32). Recent studies have proven the existence of distinct monocyte subsets and they have critical roles in the development of some rheumatic diseases such as SLE and rheumatoid arthritis (RA), and targeting monocytes is a potential treatment for those diseases (17, 33). There are also some studies focusing on the roles of monocytes in SjS, and some have shown that aberrant changes in immunophenotypes, intracellular functional pathways and epigenetics exist in SjS patients (20–24). Those outcomes suggest the key roles of monocytes in the development and progression of SjS, though the underlying mechanisms are largely elusive.

Monocytes have several subsets with functionally distinct phenotypes, and peripheral monocytes have been calcified as proinflammatory or classic monocytes (CD14^++^CD16^−^), intermediate monocytes (CD14^++^CD16^+^) and nonclassic monocytes (CD14^+^CD16^++^) (34). Though many studies have explored the roles of monocytes in human diseases, findings are inconsistent in both the immunophenotypes of monocyte subsets and their functions. The development of scRNA-seq has provided new opportunities in uncovering monocyte subsets and defining their disturbances in those diseases (14, 35–37). In the present study, we tried to identify changes in monocyte subsets and transcriptome in SjS patients *via* scRNA-seq analyses. A total of 11 monocyte subsets were identified in the scRNA-seq analyses of monocytes, and a new monocyte subset characterized by higher expressions of VNN2 (GPI-80) and S100A12 (Monocyte cluster 3) was found to increase in SjS patients. However, owing to the limited samples in current study and the minimal heterogeneity among those mononuclear phagocyte subsets, the features genes of those monocyte subsets identified in our study were not highly specific. scRNA-seq transcriptome analyses with limited number of cells or samples could undoubtedly result in high difficulty in defining unique subpopulations with specific features genes. Therefore, the changes in monocyte subsets among SjS patients still need to be explored by further scRNA-seq analyses of larger number of samples. These studies may provide new perspectives in the landscape of mononuclear phagocytes and uncover the potential key pathogenic subset in SjS.

This study suggested that IFN-related signaling and virus infection-associated pathways were key up-regulated pathways in the monocytes of SjS patients and they were involved in SjS pathogenesis. Currently, the role of IFN-α pathway in SjS pathogenesis has long been clearly defined, and therapies targeting IFN-α may be a candidate treatment strategy for SjS (38–40). Besides, there are some published literatures which could confirm the up-regulation of IFN-related signaling in the monocytes of SjS patients. A study by Brkic et al. reported that type I IFN inducible genes such as IFI44L, IFI44, IFIT3, LY6E and MX1 were systematically up-regulated in monocytes of SjS patients and were associated with high disease activity (41). Wildenberg et al. also reported that there was an upregulation of IFN-related genes such as IFI27, IFITM1, IFIT4, and IFI44 in monocytes of SiS patients (42). Sialic acid binding Ig like lectin 1 (Siglec-1), a biomarker of the activation of type I IFN pathway, was highly expressed in monocytes of SjS patients and was positively correlated with the EULAR Sjögren’s Syndrome Disease Activity Index (ESSDAI) score (43). Pertovaara et al. found increased cytokine-induced STAT1 activation in monocytes of SjS patients *via* flow cytometry (44). Data from another 2 studies also supported the up-regulation of molecules related to the activation of IFN-related signaling in the monocytes of SjS patients (45, 46). Therefore, there is good evidence supporting the up-regulation of IFN-related signaling in the monocytes of SjS patients.

Virus infection such as Epstein-Barr virus (EBV) infection has long been studied as an important environmental risk factor of SjS, but definite conclusion on its pathogenic role in SjS is still lacking (47–50). The findings from this study support virus infection as an important player in SjS pathogenesis. However, the molecular mechanisms underlying the pathogenic roles of virus infection in SjS are still not clear, and need to be elucidated in future studies. Apart from IFN-related pathways and virus infection-associated pathways, neutrophil activation-associated pathways were also identified to be up-regulated pathways in the monocytes of SjS patients. There is accumulating evidence implicating those neutrophils as key players in the pathogeneses of autoimmune or rheumatic diseases (51–53). The hyperactivation of neutrophils have been implicated in the pathogeneses of rheumatic diseases such as systemic lupus erythematosus (SLE) (54–56). An early research reported that neutrophil adhesion was enhanced in SjS patients, which indicated an increased activation of neutrophils in SjS patients (57). The up-regulation of neutrophil activation-associated pathways in the monocytes of SjS patients suggests that a possible role of monocytes-neutrophils cross-talk in the pathogenesis of SjS. Previous studies have revealed that monocytes can mediate neutrophil activation *via* multiple distinct mechanisms and is involved in diseases such as SLE (58–60). However, studies focusing on the roles of neutrophil activation or monocytes-neutrophils cross-talk in SjS are still limited, and further studies are needed.

Tumor Necrosis Factor-Related Apoptosis Inducing Ligand (TRAIL/TNFSF10) is a key cytokine of the TNF superfamily, and has significant roles in regulating immunity. Previous studies have identified abnormal changes in TNFSF10 (TRAIL) among patients with distinct autoimmune and rheumatic diseases such as SLE (61–63). Another study found that TRAIL^+^ monocytes played critical roles in lung damage (64). However, the roles of TNFSF10 ^high/+^ monocytes in common autoimmune and rheumatic diseases have not been clearly defined. In present study, the increased expression of TNFSF10 (TRAIL) in the monocytes of SjS patients was identified by both scRNA-seq transcriptome analyses (**Figure 2B**) and bulk transcriptome analyses of monocytes (**Figure 3A**). The outcomes above suggested TNFSF10 ^high/+^ monocytes as a potential key player in SjS pathogenesis and a promising target for SjS treatment. Besides, more studies exploring the roles and potential mechanisms of TNFSF10 ^high/+^ monocytes in SjS development are needed.

A limitation of this study was the small size of samples of SjS patients and controls especially in the scRNA-seq transcriptome analyses. In the scRNA-seq analyses, there were only 5 SjS cases and 4 controls, which could undoubtedly cause impaired statistical power in detecting differences across distinct monocyte subsets. The total number of monocytes in the scRNA-seq analyses was also limited, which could result in suboptimal analyses of monocyte subsets and their functions. Therefore, further scRNA-seq analyses with larger sample size are recommended in future studies, which may provide much deeper insights into the pathogenesis of SjS.

In summary, this study uncovered the abnormal changes in monocyte subsets and their transcriptomic changes in SjS patients. Both scRNA-seq and bulk RNA-seq transcriptomic analyses identified increased expression of TNFSF10 (TRAIL) in monocytes among SjS patients, suggesting TNFSF10 ^high/+^ monocytes as a potential key player in SjS pathogenesis and a promising target for SjS treatment. Further work is needed to explore the roles and underlying mechanisms of monocytes in SjS development.

## Data Availability Statement

Publicly available datasets were analyzed in this study. This data can be found here: https://www.ncbi.nlm.nih.gov/geo/query/acc.cgi?acc=GSE157278 https://www.ncbi.nlm.nih.gov/geo/query/acc.cgi?acc=GSE173670.

## Ethics Statement

The studies involving human participants were reviewed and approved by the Ethics Committee of the First Affiliated Hospital of Xiamen University. The patients/participants provided their written informed consent to participate in this study.

## Author Contributions

YH, YL, and GS contributed to conception and design of the study. YH, RC, MZ, and BW analyzed the data. YH, BW, and ZL wrote the manuscript. RC, YL, and GS reviewed and edited the manuscript. All authors contributed to manuscript revision, read, and approved the submitted version.

## Funding

This work was supported by grants from the National Natural Science Foundation of China (Grant No. 82171779) to GS, and the Fujian Provincial Health Commission (Grant No. 2021GGB025) to YL.

## Conflict of Interest

The authors declare that the research was conducted in the absence of any commercial or financial relationships that could be construed as a potential conflict of interest.

## Publisher’s Note

All claims expressed in this article are solely those of the authors and do not necessarily represent those of their affiliated organizations, or those of the publisher, the editors and the reviewers. Any product that may be evaluated in this article, or claim that may be made by its manufacturer, is not guaranteed or endorsed by the publisher.
